# Postoperative Home Monitoring After Joint Replacement: Retrospective Outcome Study Comparing Cases With Matched Historical Controls

**DOI:** 10.2196/10169

**Published:** 2018-11-05

**Authors:** Homer Yang, Geoff Dervin, Susan Madden, Ashraf Fayad, Paul Beaulé, Sylvain Gagné, Mary Lou Crossan, Kathryn Wheeler, Melody Afagh, Tinghua Zhang, Monica Taljaard

**Affiliations:** 1 Department of Anesthesia and Perioperative Medicine Schulich School of Medicine Western University London, ON Canada; 2 Division of Orthopaedic Surgery University of Ottawa Ottawa, ON Canada; 3 Department of Nursing The Ottawa Hospital Ottawa, ON Canada; 4 Department of Anesthesia and Pain Medicine University of Ottawa Ottawa, ON Canada; 5 Clinical Epidemiology Program Ottawa Hospital Research Institute Ottawa, ON Canada; 6 School of Epidemiology and Public Health University of Ottawa Ottawa, ON Canada

**Keywords:** postoperative care, postoperative home monitoring, postoperative emergency department visit, postoperative readmissions, continuity of care, cost reductions, length of stay

## Abstract

**Background:**

A retrospective cohort study was conducted in patients undergoing postoperative home monitoring (POHM) following elective primary hip or knee replacements.

**Objective:**

The objectives of our study were to compare the cost per patient, readmissions rate, emergency room visits, and mortality within 30 days to the historical standard of care using descriptive analysis.

**Methods:**

After Research Ethics Board approval, patients who were enrolled and had completed a POHM study were individually matched to historical controls by age, American Society of Anesthesiology class, and procedure at a ratio 1:2.

**Results:**

A total of 54 patients in the study group and 107 in the control group were eligible for the analysis. Compared with the historical standard of care, the average cost per case was Can $5826.32 (SD 1418.89) in the POHM group and Can $9198.58 (SD 1513.59) for controls. After 30 days, there were 2 emergency room visits (3.7%) and 0 readmissions in the POHM group, whereas there were 8 emergency room visits (7.5%) and 2 readmissions (1.9%) in the control group. No mortalities occurred in either group.

**Conclusions:**

The POHM study offers an early hospital discharge pathway for elective hip and knee procedures at a 38% reduction of the standard of care cost. The multidisciplinary transitional POHM team may provide a reliable forum to minimize readmissions, and emergency room visits within 30 days postoperatively.

**Trial Registration:**

ClinicalTrials.gov NCT02143232; https://clinicaltrials.gov/ct2/show/NCT02143232 (Archived by WebCite at http://www.webcitation.org/73WQ9QR6P)

## Introduction

### Background

Postsurgical emergency department (ED) visits and readmissions within 30 days after surgical discharge led to a marked increase in expenditures [1]. In a retrospective database study of 152,783 patients undergoing major joint replacements, 5.81% (8883/152,783) patients returned to ED within 30 days, more common than 30-day readmissions of 3.42% (5229/152,783), and pain was the most frequent single diagnosis (25.75%) [2]. Often, patients return to a nonindex hospital, which is not the hospital where surgery was performed originally [3]. The costs in such cases are higher [4], as is the mortality [5]. Data on 667,796 surgical patients from the Canadian Institute for Health Information show that 18.7% of postsurgical patients visited ED within 30 days of discharge (based on Ontario, Alberta, and Yukon data) [1]. An innovative, safe clinical pathway to provide continuity of care or transitional care after surgical discharge would seem ideal both from the patient safety and cost containment perspectives. The postoperative home monitoring (POHM) pathway is feasible and provides the transitional care team to maintain direct communication with their patients after surgery. However, the cost associated with this clinical pathway or the rate of ED visits or readmissions postoperatively have not been studied previously. In this study, we hypothesize that the outcomes of POHM are comparable to historical controls and the costs are lower.

### Objectives

This study aims to descriptively compare the rates of 30-day readmissions, number of ED visits, and total costs between POHM patients and historical controls.

## Methods

This study protocol was approved by the Research Ethics Board. Data from patients who completed the POHM study were collected, and historical controls were selected, matched in 2:1 ratio to POHM cases by age in deciles, American Society of Anesthesiology class, and procedure. Then, potentially matched controls between January 2010 and December 2012 were identified by Medical Records, and the actual control charts were selected by the RANDBETWEEN function in Microsoft Excel. The cost analysis was conducted by the hospital Finance Department as per the provincial protocols for case costing.

Outcomes were predefined and unchanged during the trial. We compared the rates of postoperative 30-day mortality, readmissions, ED visits, and the total costs between the groups.

We used descriptive statistics (mean [SD] or n [%]) to describe the preoperative and predischarge characteristics of participants. Furthermore, cases and controls were compared using descriptive statistics.

## Results

A total of 54 POHM patients (recruited between April 17, 2014 and August 31, 2015) and 107 control patients (January 2010 and December 2012) were eligible for this study. Table 1 shows the demographic characteristics and outcomes for the 2 groups. For one of the cases, an American Society of Anesthesiologists class 1, only one control was found. No 30-day postoperative mortality occurred in the controls or cases. The 30-day postoperative ED visits were 3.7% (2/54) and 7.5% (8/107) in the POHM group and controls, respectively. There were two 30-day postoperative readmissions among the controls and none among the POHM cases. Table 2 shows the direct, indirect, and total costs between the cases and controls. The average total costs were Can $5826.32 (SD 1418.89) for cases and Can $9198.58 (SD 1513.59) for controls.

**Table 1:** The case-control demographics and 30-day outcomes, postoperative home monitoring Part 2.

Variables		Postoperative home monitoring, (n=54)	Controls, (n=107)
Age, mean (SD)		61.4 (8.3)	61.9 (8.5)
Body mass index, mean (SD)		27.5 (4.0)	30.7 (6.2)
High blood pressure, n (%)		15 (27.8)	38 (35.5)
Type II diabetes mellitus, n (%)		3 (5.6)	12 (11.2)
Hypercholesterolemia, n (%)		14 (25.9)	28 (26.2)
Pain >3 mo requiring treatment, n (%)		54 (100)	96 (89.7)
Current smoker, n (%)		3 (5.8)	10 (9.4)
**Anesthesia type, n (%)**			
	Spinal	50 (92.6)	91 (85.0)
	General	4 (7.4)	16 (15.0)
30-day emergency room visit, n (%)		2 (3.7)	8 (7.5)
30-day readmissions, n (%)		0	2 (1.9)
30-day mortality, n (%)		0	0

**Table 2:** Indirect and direct costs in cases and controls, postoperative home monitoring Part 2.

Type of cost^a^	Postoperative home monitoring (n=54), Mean (SD)	Controls (n=107), Mean (SD)
Variable direct labor^b^	1277.79 (152.78)	2586.62 (601.84)
Variable direct material**-**general supplies^c^	563.22 (58.37)	637.26 (120.47)
Variable direct other^d^	101.90 (26.88)	162.94 (44.19)
Variable direct material, patient-specific supplies^e^	2373.63 (1368.88)	2724.86 (1170.15)
Fixed direct labor^f^	192.44 (21.98)	372.46 (117.92)
Fixed direct other—sundry^g^	12.02 (0.94)	6.05 (16.95)
Fixed direct building, equipment, and grounds^h^	450.05 (30.43)	247.61 (47.11)
Variable indirect^i^	626.2 (57.86)	1764.3 (412.77)
Fixed indirect^j^	229.07 (23.5)	696.48 (190.96)

^a^Cost structure in use in the province of Ontario.

^b^Nurses, lab technicians, social workers, etc.

^c^Food, dressings, etc.

^d^Contracted laundry service.

^e^Nonward stock drugs, prostheses, etc.

^f^Clerical and management staff in clinical areas.

^g^Insurance, travel expenses.

^h^Renovation, equipment maintenance contracts, including software.

^i^Clerical human resources, records, housekeeping etc.

^j^Staff in overhead areas.

## Discussion

This study shows that the 30-day readmission or ED visit rates were comparable, if not lower, between POHM and historical cohorts. Conversely, the costs were lower. Based on the current literature, for hip or knee replacements, one would expect 2%-5% postoperative complication or readmission rates [6,7]. In other words, 95%-98% of patients would be safe to be discharged when surgically ready. With the advances in surgical techniques, anesthetic management, and postoperative analgesia, we believe that earlier discharge after surgery is becoming more feasible and acceptable. As the technology evolves, the POHM infrastructure will be able to capitalize on more sophisticated monitoring, including the rapidly evolving “wearables.” The POHM solution is not expected to change complication rates but with reliable wireless connectivity, real-time interactions with patients are feasible. Such continuity of care would allow a clinician to determine when a patient could be managed at home, return to a nonindex hospital, or return to the index hospital expeditiously, thereby making earlier postsurgical discharge safer with better patient satisfaction.

Postoperative follow-up phone calls have been implemented in many centers. However, little evidence exists that follow-up phone calls by themselves reduce postdischarge readmission rates or ED visits [8-10]. Of various measures that mitigate postdischarge readmissions, continuity of care by physicians who treated patients prior to admission is the most important factor in reducing readmissions [11,12]. The model of care in this study supported the patient after discharge with a multidisciplinary team, including surgeons who had operated on patients. We believe that the model of care is a crucial element in supporting patients after discharge.

The results of this study were viewed by the hospital as an important finding and led our hospital to partner with the Ontario TeleHealth Network. The cost associated with the POHM technology (hardware and software) is expected to drop further in the future. In addition, the ability to scale up; to maintain updates, patient privacy, confidential data repository; to add other devices onto the system; and to negotiate pricing by bulk has increased the ease of application of POHM.

This study has some limitations. Retrospective historical data were used as controls but conducting a concurrently controlled study was not feasible. Because the sample size was small, we could not draw the statistical significance of differences in 30-day ED visits or readmissions, although a trend of higher rates in the control group was observed. There is potential of missing the 30-day returns in the control group if a patient did not return to our hospital or was readmitted at another hospital. Nevertheless, the trend being already higher in the control group would suggest that if there were a bias, it would have been an underdocumenting of the 30-day mortality, readmissions, or ED visits in the control group. In addition, the cost tracking over the 2 periods in the chart audit was based on the same provincial methodology and with a relatively stable inflation rate, we believe the true cost differences are reflected in our comparisons. The physician costs both in terms of consults, both in patients with longer length of stay and in patients with 30-day ED visits or readmissions, were not tracked. As alluded to earlier, 30-day ED visits or readmissions in the control group to nonindex hospitals were not tracked and their costs, therefore, are not included. Nevertheless, the bias would have been in favor of the control group.

In conclusion, we believe that POHM is a new paradigm of postacute care model for surgical recovery, providing better surgical access by further reducing the length of stay, 30-day ED visits by providing continuity of care and addressing patient concerns, and 30-day readmission rates by stratifying postdischarge management at home, at a nonindex hospital, or return to the index hospital.

## Abbreviations

ED: emergency department
POHM: postoperative home monitoring
